# Safety and Tolerability of Fractional CO_2_ Lasers for Facial Rejuvenation Across Fitzpatrick Skin Phototypes I–IV: Retrospective Cohort Study

**DOI:** 10.1111/jocd.70917

**Published:** 2026-05-12

**Authors:** Ayooluwatomiwa I. Oloruntoba, Michelle Rodrigues

**Affiliations:** ^1^ Alfred Health Melbourne Victoria Australia; ^2^ Monash University School of Public Health and Preventative Medicine Melbourne Victoria Australia; ^3^ University of Melbourne Melbourne Victoria Australia; ^4^ Royal Children's Hospital Melbourne Victoria Australia; ^5^ Chroma Dermatology, Pigment and Skin of Colour Centre Melbourne Victoria Australia

## Abstract

**Background:**

Fractional CO_2_ laser is an effective treatment for skin rejuvenation, although pigmentary complications remain a clinical consideration. Newer generation fractional CO_2_ laser systems include pulse modes such as HighPulse (HP), which deliver high energy over a shortened dwell time, reducing the duration of energy delivery per microbeam with the aim of limiting thermal diffusion beyond the ablation zone.

**Methods:**

We conducted a single‐center retrospective study of 38 patients (Fitzpatrick skin phototypes I–IV) treated with fractional CO_2_ laser using HP mode for facial rejuvenation. Pain scores and adverse events were recorded, stratified by skin type and anesthetic use.

**Results:**

Mean pain score was 4.8, with no statistically significant difference between patients who received topical anesthetic (LMX4) and those who did not. Pain scores did not differ significantly across skin phototypes I–IV. Erythema occurred in 50% of patients and edema in 24%, all of which were transient. No cases of blistering, post‐inflammatory hyperpigmentation, or scarring were documented in routine clinical records, although the true incidence may be underestimated. Erythema rates did not differ significantly by anesthetic use.

**Conclusions:**

In this preliminary cohort, the HP fractional laser was associated with transient short‐term adverse effects and no documented pigmentary complications among patients with Fitzpatrick skin types I–IV. These findings are descriptive and are not generalizable to Fitzpatrick phototypes V–VI, which were not represented.

## 1 Introduction

Fractional carbon dioxide (CO_2_) laser is an established treatment for skin rejuvenation, improving fine lines, texture, and dyschromia through controlled dermal ablation and stimulation of collagen remodeling [1]. The development of fractional laser technology has reduced the risks associated with fully ablative resurfacing by delivering energy in microscopic treatment zones, allowing adjacent untreated skin to facilitate rapid healing [2]. Newer‐generation fractional CO_2_ lasers can also be delivered in modes that reduce the thermal tail, defined as the zone of residual heat that extends beyond the ablation site [3]. This is intended to minimize thermal damage to surrounding tissue [4].

These considerations are particularly relevant for individuals at higher risk of pigmentary sequelae, in whom thermal injury to the epidermis can trigger post‐inflammatory hyperpigmentation (PIH) and scarring [5, 6, 7]. Despite advancements in laser safety, concerns regarding pigmentary complications may contribute to hesitancy toward fractional CO_2_ laser procedures, with clinicians often favoring non‐ablative alternatives perceived as lower risk [8, 9].

Current literature remains limited regarding the real‐world tolerability of fractional CO_2_ lasers in phototypes at higher risk of pigmentary complications. Data on modern low‐thermal diffusion pulse modes are sparse, particularly beyond Fitzpatrick skin types I–IV [10, 11].

This retrospective study evaluates the tolerability and side effect profile of fractional CO_2_ laser resurfacing for facial rejuvenation in patients with Fitzpatrick skin types I–IV. By assessing patient‐reported discomfort and clinician‐recorded adverse events, we aim to characterize short‐term tolerability within this phototype range. Fitzpatrick skin types V and VI were not represented and are outside the scope of this analysis. Given the retrospective design, small sample size, and absence of Fitzpatrick phototypes V and VI, this study is intended as a preliminary, descriptive analysis and does not aim to provide generalizable conclusions.

## 2 Methods

This was a single‐center retrospective observational study based on routinely collected clinical data from a private dermatology clinic, reflecting routine clinical workflows rather than a prospectively designed research protocol. Patients who underwent fractional CO_2_ laser treatment for facial rejuvenation between January 2021 and January 2025 were included. The study was approved by the Human Ethics Review Board on 29 July 2025. Data collection and analysis were performed between August and October 2025. Written informed consent was obtained from all participants, permitting the use of their de‐identified clinical data for research.

Inclusion criteria included treatment with fractional CO_2_ laser for rejuvenation purposes, including fine lines, wrinkles, and textural irregularities. Exclusion criteria were treatment for non‐rejuvenation indications, use of other energy‐based devices during the same course, or incomplete documentation.

Collected variables included age, sex, Fitzpatrick skin phototype, treatment area, laser mode, stacking level, topical anesthetic use, and clinical outcomes. Pain was assessed using a 0–10 numeric rating scale immediately post procedure, consistent with routine clinical practice. 0 represented no pain and 10 the worst imaginable pain. Where used, topical anesthesia consisted of LMX4 (4% liposomal lidocaine), applied for 45 min without occlusion.

Phototype‐based analyses were performed across Fitzpatrick skin types I–IV. Fitzpatrick skin types V and VI were not represented in this cohort.

Adverse events were recorded through routine clinician documentation during treatment and follow‐up, without use of a standardized grading scale or consistent photographic assessment protocol, consistent with standard clinical practice in this setting. Adverse events were recorded as erythema, edema, post‐inflammatory hyperpigmentation, and hypopigmentation. Erythema and edema were assessed immediately after treatment. Erythema was defined as transient if it resolved within 72 h, and edema within 48 h. Documentation was based on clinician observations at the time of treatment and follow‐up. All data were de‐identified prior to analysis. Outcome assessment was not blinded due to the retrospective nature of the study.

All treatments were performed using the SmartXide Punto fractional CO_2_ laser system (Deka, Florence, Italy), which incorporates Pulse Shape Design (PSD) technology. The device includes three emission modes: SmartPulse (SP), DEKAPulse (DP), and HighPulse (HP). This study exclusively used the HighPulse mode, which is designed to deliver high energy over shorter pulse durations, with the aim of limiting collateral thermal diffusion. Dwell time refers to the duration of laser energy delivery per microbeam. Longer dwell times increase conductive heat spread beyond the ablation column, contributing to the “thermal tail,” defined as the zone of residual injury surrounding the treated area.

### 2.1 Statistical Analysis

Descriptive statistics were used to summarize patient characteristics and treatment outcomes. Categorical variables were analyzed using chi‐squared or Fisher's exact tests. Pain scores were assessed for normality and compared using the Mann–Whitney *U* test or Kruskal–Wallis test, with Dunn's test and Bonferroni correction applied for post hoc comparisons. All analyses were conducted using Stata 18.0 (StataCorp, College Station, TX) and GraphPad Prism 10.0 (GraphPad Software, San Diego, CA). A two‐sided *p*‐value of < 0.05 was considered statistically significant.

Given the small sample size and low event frequency, multivariable modeling was not performed. The study was not powered to detect rare complications or small differences between subgroups. Accordingly, nonsignificant findings should not be interpreted as evidence of equivalence, and residual confounding cannot be excluded.

## 3 Results

**FIGURE 1:**
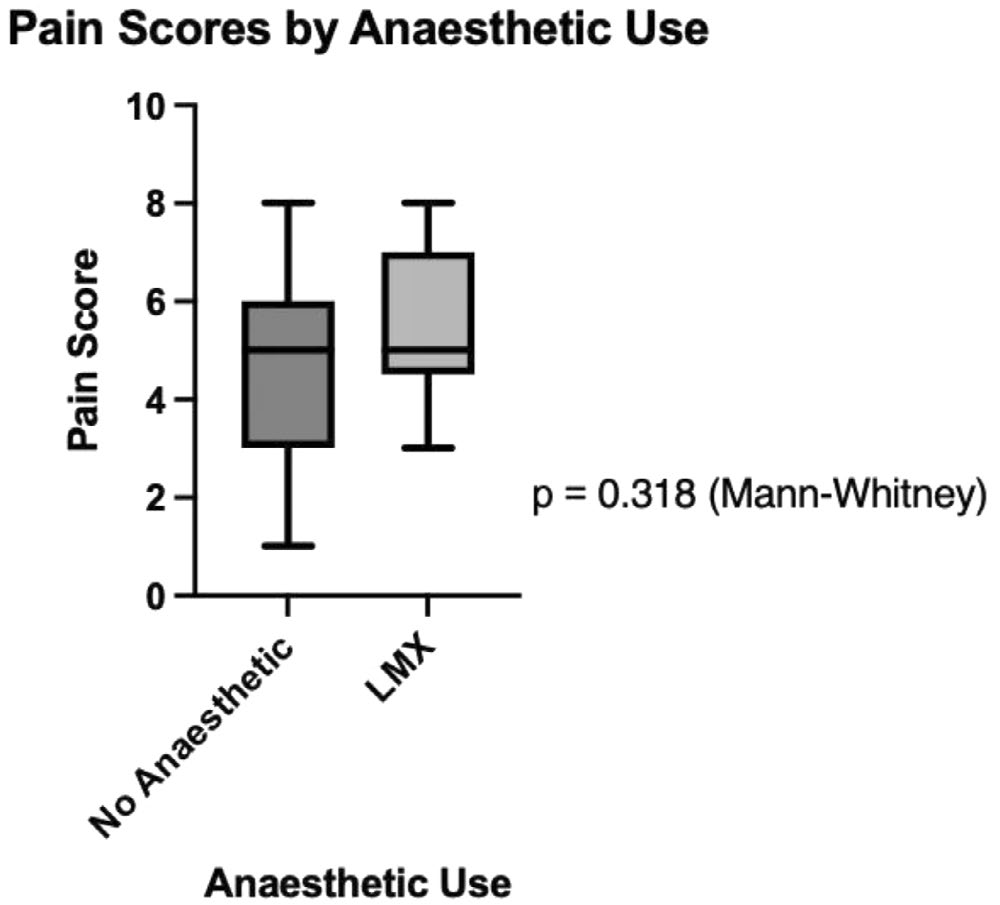
Pain scores by anesthetic use. Comparing patient‐reported pain scores during fractional CO_2_ laser treatment between those who received topical LMX4 and those who received no anesthetic.

**FIGURE 2:**
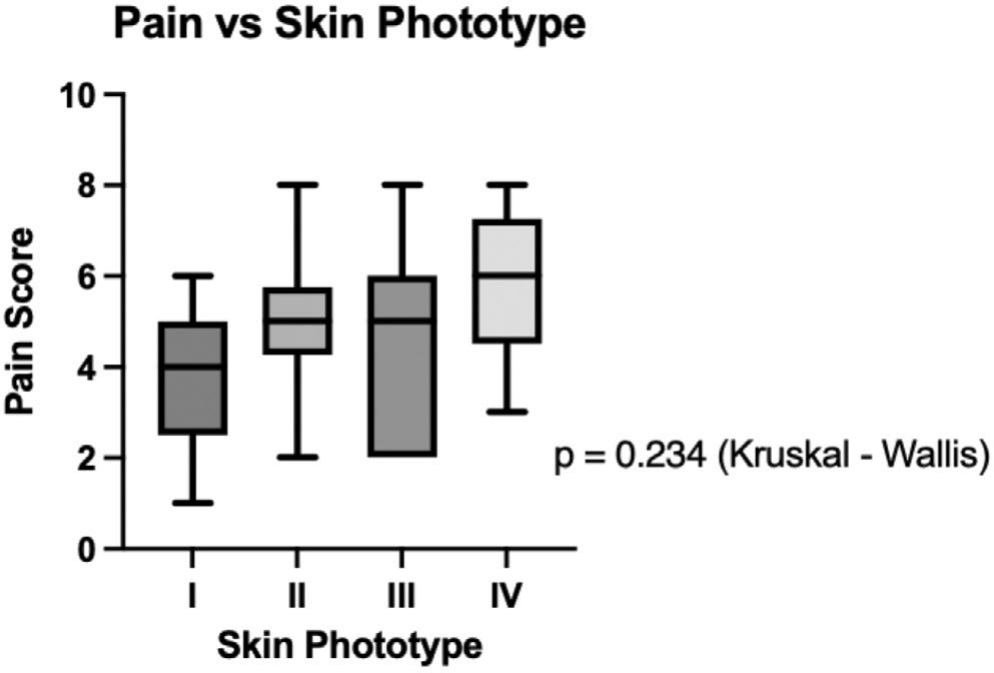
Pain scores by Fitzpatrick skin phototype. Distribution of patient‐reported pain scores (0–10) across Fitzpatrick skin types I–IV following fractional CO_2_ laser treatment using HP mode.

A total of 38 patients underwent fractional CO_2_ laser treatment using the HP mode for facial rejuvenation. The cohort included 35 females and 3 males, with a mean age of 51.9 years. Fitzpatrick skin types I–IV were represented (Table 1). The mean reported pain score across the cohort was 4.8. No significant difference in pain scores was observed between patients who received topical anesthetic and those who received no anesthesia (*p* = 0.318; Figure 1). Pain scores did not differ significantly across Fitzpatrick skin phototypes (*p* = 0.234; Figure 2).

**TABLE 1:** Summary of patient demographics, treatment characteristics, and reported side effects in CO_2_ laser rejuvenation cohort (*n* = 38).

Category	Value
Complete datasets	38
Sex distribution	35 female, 3 male
Average age	51.8 years
Treatment sites	38 single site
Skin type distribution	Type I: 8, Type II: 12, Type III: 12, Type IV: 6
Reported side effects	
Erythema	19 patients
Edema	9 patients
Blistering	0 patient
Discoloration	0 patient
Laser settings	
Laser mode	HP: 38
Anesthetic used	LMX: 9, None: 29

Transient erythema was the most common adverse event, documented in 19 patients (50%), followed by transient edema in nine patients (24%). No cases of blistering, post‐inflammatory hyperpigmentation, or hypopigmentation were documented in routine clinical records. However, outcome capture was limited by non‐standardized follow‐up and lack of systematic photographic assessment. All reported erythema resolved within 72 h and all edema within 48 h (Table 1). No prolonged adverse events were documented.

**FIGURE 3:**
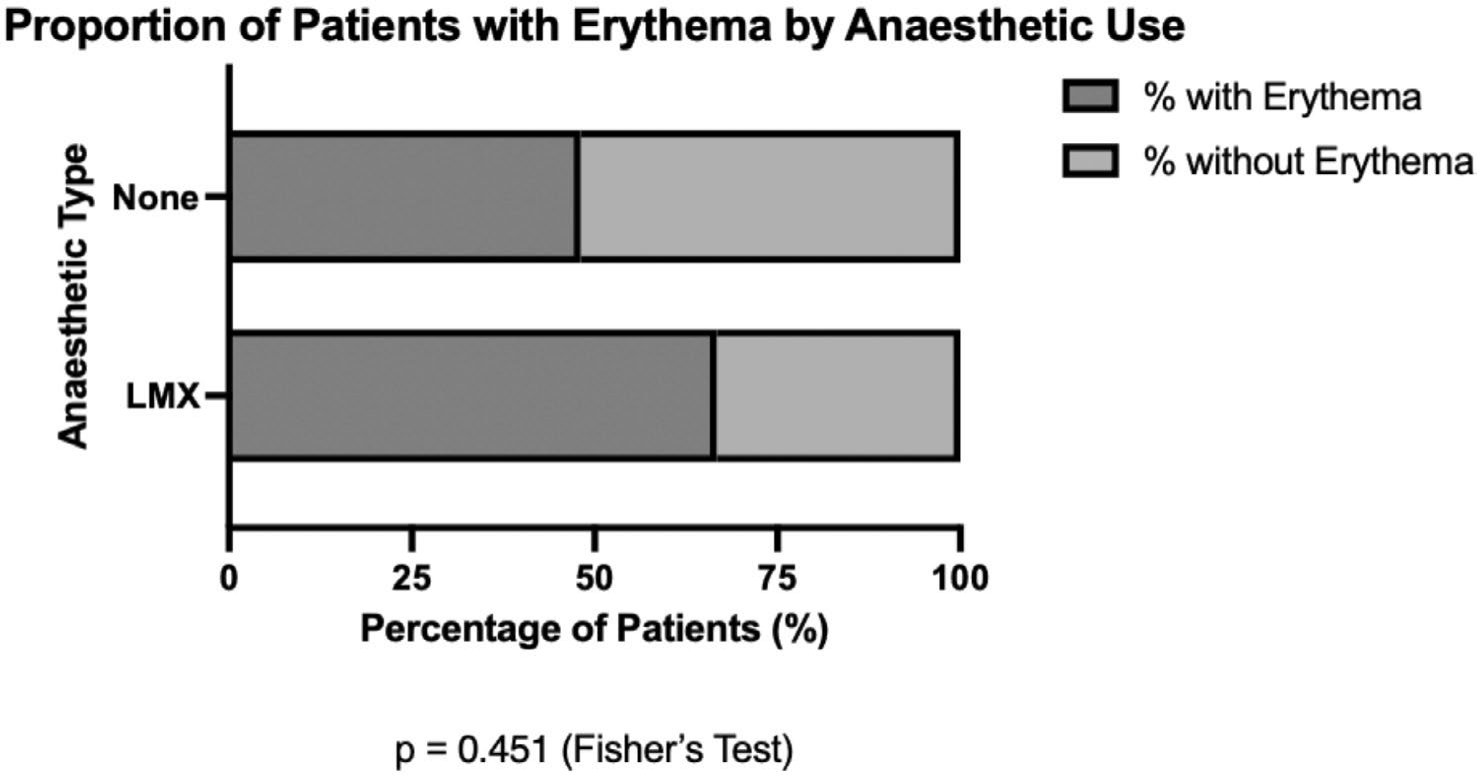
Proportion of patients with erythema by anesthetic use. Percentage of patients who developed erythema following fractional CO_2_ laser treatment, stratified by anesthetic use.

**FIGURE 4:**
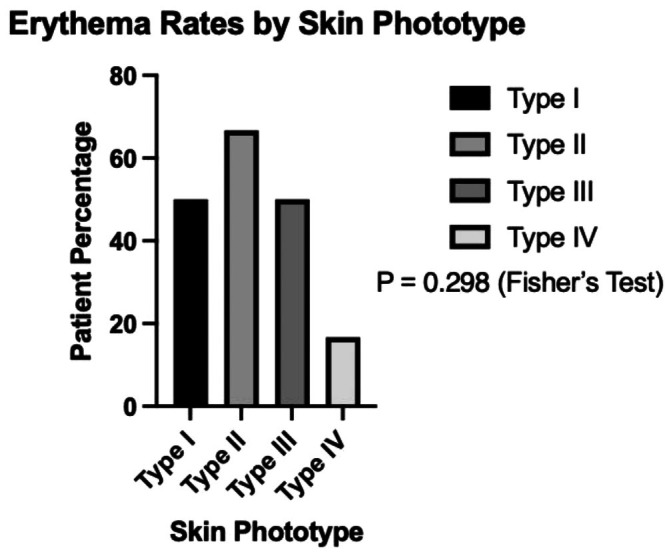
Erythema rates by Fitzpatrick skin phototype. Percentage of patients who developed erythema following fractional CO_2_ laser treatment, stratified by Fitzpatrick skin type.

No statistically significant association was observed between topical anesthetic use and the presence of erythema (*p* = 0.451; Figure 3). Erythema rates did not differ significantly across Fitzpatrick skin phototypes (*p* = 0.298; Figure 4).

## 4 Discussion

In this study, pain scores following fractional CO_2_ laser treatment using HP mode for facial rejuvenation were moderate overall, with a mean of 4.8, and did not differ significantly by topical anesthetic use or Fitzpatrick skin phototype. However, given the small sample size and low event frequency, the study was underpowered to detect modest differences between subgroups. Accordingly, the absence of statistically significant differences is most likely attributable to limited statistical power (Type II error) rather than true biological equivalence. These findings should therefore be interpreted as descriptive rather than evidence of comparable responses across phototypes or treatment conditions.

In this cohort, topical LMX4 use was not associated with a reduction in reported pain scores during fractional CO_2_ laser treatment. However, this finding should be interpreted with caution given the potential for confounding by indication, whereby patients anticipated to experience greater discomfort or undergoing more intensive treatment may have been more likely to receive topical anesthesia. While topical anesthetics are commonly assumed to improve tolerability during laser procedures, pain remains a highly subjective outcome influenced by numerous individual and procedural factors [12]. Prior studies evaluating noninvasive anesthetic strategies in fractional CO_2_ laser resurfacing have reported variable results, with several suggesting that topical agents alone may provide limited analgesia and that adjunctive measures may be required for optimal pain control [13, 14, 15]. In the absence of clear evidence favoring one approach over another, anesthetic strategies should be individualized based on patient tolerance and treatment context.

Erythema was the most frequently documented adverse event in this cohort. Assessment of erythema may be influenced by skin pigmentation as erythematous changes can be less visually apparent in more pigmented skin, a limitation that has been documented in clinical practice and dermatologic assessment [16, 17, 18]. Accordingly, detection of erythema based on routine clinical observation may be subject to under‐recognition in patients with darker skin phototypes.

In this study, no statistically significant differences in erythema rates were observed by Fitzpatrick skin phototype or topical anesthetic use. However, given the small sample and low event rates, the analysis was likely underpowered to identify subgroup differences. The absence of statistically significant findings should therefore not be interpreted as evidence of equivalent responses.

In this cohort, no cases of prolonged erythema, edema, or post‐inflammatory pigmentary change were documented in routine clinical records following fractional CO_2_ laser treatment. This may underestimate true incidence due to the retrospective design, lack of standardized assessment, and absence of systematic photographic follow‐up. Previous studies of fractional CO_2_ laser resurfacing have reported higher rates of pigmentary complications and prolonged erythema, particularly in Fitzpatrick skin types III–VI [6, 7, 19, 20, 21].

Given the single‐arm retrospective design, causal attribution of these findings to the HP mode cannot be made, and direct comparison with other fractional CO_2_ laser configurations is not possible. The absence of documented pigmentary complications in this cohort does not establish causality but may justify further evaluation of pulse configuration parameters in controlled comparative studies.

This study has several limitations that directly constrain the interpretation and clinical applicability of its findings. As a retrospective analysis based on routinely collected clinical data, outcome assessment was not standardized and did not include validated pain instruments such as a visual analogue scale, formal adverse event grading systems, consistent photographic documentation, or blinded evaluation. These methodological constraints reduce measurement precision, increase susceptibility to reporting and inter‐observer variability, and preclude robust comparison with prospectively designed studies.

The small sample size further limits inferential interpretation. With only 38 patients and a low frequency of adverse events, the study was underpowered to detect uncommon complications or true differences between subgroups, including across Fitzpatrick skin phototypes. As a result, the absence of statistically significant differences should not be interpreted as evidence of equivalence, and the risk of Type II error is substantial.

Cohort composition also limits generalizability. Male patients were under‐represented, Fitzpatrick skin type IV accounted for a minority of the cohort, and skin types V and VI were not included. Accordingly, conclusions cannot be extrapolated beyond Fitzpatrick skin types I–IV and safety in darker phototypes remains uncharacterized in this study.

Finally, treatment effectiveness, patient satisfaction, and longer‐term outcomes were not evaluated. While the present analysis provides descriptive short‐term tolerability data, it does not inform clinical effectiveness, durability of results, or patient‐reported benefit. Future research should address these limitations through prospective study designs incorporating larger and more diverse cohorts, particularly including Fitzpatrick skin phototypes IV–VI. Standardized outcome measures, such as validated pain scales and erythema grading systems, should be implemented alongside blinded assessment and serial photographic documentation to improve measurement precision. Regular follow‐up is required to capture delayed pigmentary complications. Comparative studies evaluating different pulse modes and treatment parameters are also needed to determine whether HP configurations meaningfully influence safety and tolerability outcomes, particularly in relation to post‐inflammatory hyperpigmentation risk.

In this retrospective cohort, fractional CO_2_ laser treatment using HP mode was associated with transient adverse effects, and no pigmentary complications were documented in routine clinical records among patients with Fitzpatrick skin types I–IV, although this may underestimate true incidence given the retrospective, non‐standardized nature of outcome assessment. These findings provide descriptive short‐term tolerability data within this cohort. Given the single‐arm design, limited sample size, and absence of darker phototypes, conclusions regarding causality, comparative safety, or applicability beyond Fitzpatrick skin types I–IV cannot be drawn. Prospective controlled studies are required to define the safety and tolerability of fractional CO_2_ laser treatment across broader skin type populations.

## Author Contributions

A.O.: study design, data curation, formal analysis, and writing (original draft preparation). M.R.: conceptualization, methodology, supervision, and writing (review and editing). All authors reviewed and approved the final version of the manuscript.

## Ethics Statement

This study was approved by an institutional Human Research Ethics Committee (approval date: 29 July 2025) and was conducted in accordance with the principles of the Declaration of Helsinki.

## Consent

Written informed consent was obtained from all participants, permitting the use of their de‐identified clinical data for research purposes.

## Conflicts of Interest

A/Prof Michelle Rodrigues is a key opinion leader for DEKA.

## Data Availability

The data that support the findings of this study are available on request from the corresponding author. The data are not publicly available due to privacy or ethical restrictions.
